# A revision of the genus *Muricea* Lamouroux, 1821 (Anthozoa, Octocorallia) in the eastern Pacific. Part I: Eumuricea Verrill, 1869 revisited

**DOI:** 10.3897/zookeys.537.6025

**Published:** 2015-11-18

**Authors:** Odalisca Breedy, Hector M. Guzman

**Affiliations:** 1 Centro de Investigación en Estructuras Microscópicas, Centro de Investigación en Ciencias del Mar y Limnología, Universidad de Costa Rica. P.O. Box 11501-2060, Universidad de Costa Rica, San José, Costa Rica; 2 Smithsonian Tropical Research Institute, P.O. Box 0843-03092, Panama, Republic of Panama

**Keywords:** Alcyonacea, *Astrogorgia*, Cnidaria, eastern Pacific, *Eumuricea*, *Muricea*, *Leptogorgia*, plexaurid gorgonian, soft corals, *Swiftia*, taxonomy

## Abstract

*Muricea* is an amphi-American genus. Verrill proposed dividing the species from the Pacific Ocean into three genera and established the genus *Eumuricea* for five eastern Pacific species with tubular calyces. *Eumuricea* is basically characterized by colonies with elongate, cylindrical calyces with truncate margins and star-like opercula, and the occurrence of unilateral spinous spindles. According to these characteristics, *Eumuricea* does not show enough difference from *Muricea* to be treated as a separate genus. Original type material of *Eumuricea* was morphologically analysed and illustrated using optical and scanning electron microscopy. We conclude that the eastern Pacific species should be placed in the genus *Muricea* and form a group characterised by tubular calyces that comprises four species at present, *Muricea acervata*, *Muricea hispida*, *Muricea squarrosa*, and *Muricea tubigera* and a dubious species *Muricea horrida*. Lectotypes were designated for *Muricea squarrosa* and *Muricea hispida* to establish their taxonomic status. The genus *Eumuricea* has also been misunderstood by former authors who erroneously assigned species to it. For these species we propose new combinations: *Swiftia pusilla*, *Astrogorgia splendens* and *Astrogorgia ramosa*.

## Introduction

*Muricea* Lamouroux, 1821 is an amphi-American genus with representatives in the western Atlantic and the eastern Pacific (Verrill 1869a, Bayer 1961). The eastern Pacific species were revised by Verrill (1869a) in his paper “Notes on Radiata”, where three subdivisions for the genus were proposed (Verrill 1869: 449-450), and considered by him as more than subgeneric value – two groups for the eastern Pacific species: *Eumuricea* and *Muricea* (family Plexauridae), and a third group for the Indian Ocean: *Muricella* Verrill, 1869a (presently in the family Acanthogorgiidae).

The genus *Muricella* includes species with rather thin coenenchyme, filled with long spindles, low subconical calyces arising from between the large spindles, usually standing at right angles from the axis, and covered with much smaller and shorter spindles (Verrill 1869a). As defined by Verrill, many other species could fit in this genus. Fabricius and Alderslade (2001) gave a more precise description: *Muricella* has planar fans, often net-like, and at least two-thirds of a meter tall. The polyps are short and dome-shaped, non-retractile. The sclerites are mainly spindles and small capstan, and rods. The colonies could be brown, yellow, pink and white. The polyp colour may contrast to the coenenchyme colour. The zoogeographic distribution of this genus is not clear (Fabricius and Alderslade 2001).

The eastern Pacific *Eumuricea* and *Muricea* were separated, according to Verrill (1869a), by differences in the calyx structures. He established *Eumuricea* for species with “tubular calyces, without a prolonged lower border and not bilabiate; and *Muricea* for species with more or less prominent calyces, with a more or less prolonged lower border, and bilabiate”. Hickson (1928) commented on the vaguely-defined characters used by Verrill to separate the *Muricea* groups, but did not proposed a better alternative. He pointed out, defining *Muricea* based on “bilabiate calyx” and “one-sided” spindles is inappropriate, especially because the terms were not properly explained. He said that in most of the species of *Muricea* the calyx is bent more or less upwards, a characteristic that cannot be observed in well preserved specimens, and also that the “one-sided” spindles are present in most of the species and there is a large variety of them. For these reasons Hickson (1928) considered that the only valid character to separate the groups is the tubular calyces in *Eumuricea*, but he did not refer to that as a character of generic value.

*Eumuricea* was proposed by Verrill (1869a) for four species in the YPM collection (during his time) from various localities along the eastern Pacific; he also transferred *Muricea horrida* Möbius, 1861 (from Perú) to *Eumuricea*. The lack of good illustrations and clear definitions has historically led authors to assign species erroneously to this genus (e.g. Riess 1929 (in Kükenthal 1919 and 1924), Nutting 1909, Thomson and Simpson 1909, Thomson (1927). Riess (1929) described *Eumuricea atlantica* from Jamaica; however, her description was not consistent with the characters proposed for *Eumuricea*. Deichmann (1936) made reference to this same species without any comment about its status. Later, Bayer (1961) clarified the status of *Eumuricea atlantica* as being a species of *Muricea*. Thomson (1927) listed two species for the eastern Atlantic *Eumuricea rugosa* and *Eumuricea rigida*; however, Deichmann (1936) doubted that these species belonged to this genus. Grasshoff (1992) synonymised *Eumuricea rugosa* with *Leptogorgia ruberrima* (W. Koch, 1886), and Ofwegen (2014) assigned *Eumuricea rigida* to the genus *Thesea*. Three other species: *Eumuricea splendens* Thomson & Simpson, 1909, *Eumuricea ramosa* Thomson & Simpson, 1909, and *Eumuricea pusilla* Nutting 1909, were included in this genus. The taxonomic status of these species is discussed here.

Other authors revisited this genus. Kükenthal (1924) published a key and a short review of the described species of *Eumuricea*. Aurivillius (1931) commented about this genus and remarked that the species described by Thomson and Simpson (1909), and by Nutting (1909) did not belong to the genus *Eumuricea*. Bayer in his 1981 key restored several genera that had been treated as junior synonyms or as subgenera by previous authors to a valid generic status, and stated the possibility of several more that would be validated when comparative studies have been completed. Thus, the genus *Eumuricea* was retained and separated from *Muricea*.

As it has been addressed before (e.g. Breedy and Guzman 2011), the lack of well-defined characters, good specimen and sclerite illustrations, and holotype designations in the original publications have made it difficult to recognise with certainty the species of *Muricea* (as is the case in other eastern Pacific genera).

This research represents the first part of the fifth review in a series proposed to evaluate the gorgonian genera historically reported for the shallow eastern Pacific waters. The second part will treat the genus *Muricea* Lamouroux, 1821 *sensu stricto*. Previous reviews dealt with *Pacifigorgia* Bayer, 1951 (Breedy and Guzman 2002), *Leptogorgia* Milne Edwards & Haime, 1857 (Breedy and Guzman 2007) and *Eugorgia* Verrill, 1868 (Breedy et al. 2009), in the family Gorgoniidae; and *Heterogorgia* Verrill, 1868 in the family Plexauridae (Breedy and Guzman 2011).

### Acronyms

MNHUK Museum of Natural History (former BM, British Museum), London, UK

CIEMIC Centro de Investigación en Estructuras Microscópicas, Universidad de Costa Rica

CRBMco Colección de referencia de Biología Marina Universidad Del Valle, Cali, Colombia

IMARPE Instituto del Mar de Perú, Lima, Perú

INN NAZCA Instituto de Investigaciones Marinas, Salinas, Ecuador

MCZ Museum of Comparative Zoology, Harvard University, Boston, USA

SEM Scanning Electron Microscopy

STRI Smithsonian Tropical Research Institute, Panamá

UCR Museo de Zoología, Escuela de Biología, Universidad de Costa Rica, Costa Rica

UNIANDES-BIOMMAR Universidad de Los Andes, Laboratorio de Biología Molecular Marina, Bogotá, Colombia

UPCH Colecciones Biológicas, Universidad Peruana Cayetano Heredia, Lima, Perú

USNM Museum of Natural History (former United States National Museum), Smithsonian Institution, Washington, USA

YPM Yale Peabody Museum of Natural History, New Haven, USA

ZMH Zoologisches Institut und Zoologisches Museum der Universität Hamburg, Germany

## Material and methods

The type specimens used in this study were analysed during visits to museums or acquired on loan from the BM, MCZ, USNM, and YPM. Comparative material was analysed from the collections deposited in CRBMco, IMARPE, INN and UPCH. In addition to specimens recently collected from the Pacific coast of Costa Rica and Panama deposited in the UCR and STRI. All reference material was collected by scuba diving down to 40 m in depth. The type material presented here from the western Atlantic and Indo-Pacific was the only one available to us at this time.

### Morphological study

For microscopic study, specimens were prepared for SEM following the standard protocol described in Breedy and Guzman (2002). For optic microscopy, sclerites were mounted in water or glycerine and photographed with an Olympus LX 51 inverted microscope. Sclerites of the coenenchyme and calyces are variable in size and form; the prevailing kinds are illustrated and described. Measurements of the sclerites were obtained from pictures and directly from the microscope using an optical micrometer. Length of the sclerites was measured from one tip to the other and the width was taken from the most distant points across the sclerites, reporting the largest sizes found in the samples and also, a range of variation. The diameter of the branches, branchlets, and stems are given taking in account the calyces length, the reported measurements represent the largest sizes found in the sample and in some cases, a range of variation. The mean number of calyces by cm was taken from pictures, counting the number of calyces on one side (the one showed in the picture) of each tip branch of the colony and is reported as the mean (number of calyces by cm/ number of branches measured). The limitations found in this procedure were due to the preservation state of the specimens and the number of branches. We emphasize that this measure is just a reference number and does not represent the real number of calyces/cm around the branch of each species, but is a good character to show the tendency in a species. The calyx length given corresponds to the upper parts of the branches. The colours of the colonies and sclerites are stable, and persist after fixation. Some fading is observed in dry specimens. When possible the colours of the colony alive, preserved and dry are mentioned.

Data on geographical distribution are from our personal collections, museum catalogues, and published monographs. In some cases there is just one specimen in the collection under a species name, which automatically constitutes the holotype. When needed we designated lectotypes to establish the species identity and avoiding future confusion.

### Terminology

Terminology is according to Bayer (1961) and Bayer et al. (1983). Some term modifications dealing with *Muricea* descriptions are included below.

*branched spindle*: a spindle with some of the processes much elongated and branchlike, often crooked.

*calyx*: cylindrical or wartlike projecting anthostele. In *Muricea*, it is mostly formed by the same type as the outer coenenchyme sclerites. They are arranged as a fringe around the border of the calyx with the sharp or spiny processes projecting outwards.

*calyx shelf-like*: calyx with prolonged lower border, polyp opens upwards.

*calyx tubular*: tube-like calyx with mostly even, truncate borders, polyp opens straight and distally.

*club*: monoaxial sclerite enlarged at one end, the head, and tapered at the other end, the handle. According to the modification of the head they are classified in leaf, thorn, wart or torch. *Muricea*, the heads are mostly ornamented by sharp, thorn-like or spine-like processes, and the handles are warty, or of an almost smooth surface, curved or straight.

*unilateral spinous spindles*: sclerites with asymmetrically arranged warts on the surface. Inner side with low composite warts, close together or very crowded, their processes often anastomose. Outer side with fewer cone-shaped tubercles, long projecting in some cases. Ends of the sclerites blunt, acute or both.

### Notes on morphological characters

Variation is expected in the diameter of stems and branches either in preserved or dry material, due to the preservation history of specimens. Most of the type material is dry and old (more than a century). Some specimens are deteriorated. According to Hickson (1928) the drying or preservation process can affect some characteristics, especially the calyces. However, we have observed that the tendency of the calyces to be slightly raised or prominent is kept after retraction during preservation, and it is also observed in living specimens. The calyx length and spacing vary from the larger branches to the thinner, being larger and acute, and closer placed on the branchlets and shorter, blunt, and distant on the main branches. For these reasons, we record measurements of these characters from the upper part of the branches. The sclerites that compose the outer coenenchyme and the calyx are mostly large spindles of several shapes. Bayer (1961) refers to this type of spindle as unilateral spinous and it is the term that we used here for the descriptions (defined above).

The polyp sclerites are basically rods and spindles, in most of the cases it was not possible to determine the anthocodial arrangement because of the deterioration of the type material, few type specimens were found preserved in ethanol. However, we did notice that there is no collaret and points arrangements as in other plexaurids. The sclerites are mostly placed longitudinally in irregular arrangements or in some cases in points. Although some variation is expected, the colour of sclerites and colonies is remarkably constant. Some species dye the ethanol a dark purplish colour when preserved. In this genus, colours are mostly hues of brown, and the sclerites do not present much colour variation.

## Taxonomy

### Key to distinguish genera (modified from Bayer 1981)

**Table:** 

1	Sclerites in form of spindles and capstans, with tubercular sculpture arranged in whorls, measuring less than 0.3 mm long. Anthocodial sclerites mainly flat rods forming weak or irregular collaret and points arrangements	***Leptogorgia***
–	Sclerites in form of spindles highly modified, measuring more than 0.3 mm long. Anthocodial sclerites other than flat rods forming collaret and points or variation of that arrangement	**2**
2	Coenenchyme contains unilateral spinous sclerites, polyps retract into shelf-like or tubular calyces	***Muricea***
–	Coenenchyme does not contain unilateral spinous sclerites, polyps retract into prominent or slightly raised dome-shaped polyp mounds	**3**
3	Coenenchymal sclerites mostly spindles, straight, curved, branched, heavily ornamented with complex tubercles, and prickles. Sclerites below the points may be transverse, but small and numerous, not forming distinct collaret	***Astrogorgia***
–	Coenenchymal sclerites mainly capstans, radiates and spindles, thin, sharp, with tubercles, some modified as incomplete disks, but not heavily ornamented	***Swiftia***

### Class Anthozoa Ehrenberg, 1834 Subclass Octocorallia Haeckel, 1866 Order Alcyonacea Lamouroux, 1812

#### Family PLEXAURIDAE Gray, 1859

##### Genus Muricea

Taxon classificationAnimaliaAlcyonaceaPlexauridae

Lamouroux, 1821

Muricea Lamouroux, (pars) 1821: 36; Blainville (pars) 1834: 509; Ehrenberg (pars) 1834: 134; Dana 1846: 673; Milne Edwards and Haime 1850: 142; Kölliker 1865: 135; Verrill 1868b: 411; Verrill 1869a: 418–419, 450; Studer 1887: 58; Wright and Studer 1889: 93; Gorzawsky 1908: 8; Nutting 1910: 9; Kükenthal 1919: 835; 1924: 141; Riess 1929: 383–384; Aurivillius 1931: 102–104; Deichmann 1936: 99; Bayer 1956: F210; 1959: 12; 1961: 179–180; 1981: 930 (in key); 1994: 23–24; Tixier-Durivault 1969–1970: 154; Harden 1979: 140; Hardee and Wicksten 1996: 127–128; Marques and Castro 1995: 162; Castro et al. 2010: 779.
Eumuricea (pars) Verrill 1869a: 449; Riess 1929: 397; Studer 1887: 58; Wright and Studer 1889: pl LVI; Nutting 1909: 718; Thomson and Simpson 1909: 258; Thomson 1927: 48–49; Kükenthal 1919: 836; 1924: 149–150; Riess 1919: 397–398; Aurivillius 1931: 50 (emended); Deichmann 1936: 104.
Eumuricea (Muricea) Bayer 1981: 930 (in key).

###### Type species.

*Muricea spicifera* Lamouroux, 1821, by subsequent designation: Milne Edwards and Haime 1850. [*Muricea spicifera* was later synonymised with *Muricea muricata* (Pallas, 1766) *apud* Bayer 1961: 179–180]

**Diagnosis** (based on Bayer 1961, 1994; Marques and Castro 1995; Castro et al. 2010).

Colonies planar or multiplanar, bushy, arborescent, laterally branched, pinnately branched, dichotomous or with long flexible branches without occasional branch anastomosis. Branches and branchlets upward bending almost parallel, and with about the same thickness all along, frequently with slightly enlarged tips. Coenenchyme moderately to very thick (compared to other plexaurids) with a circle of longitudinal canals surrounding the axis and dividing the coenenchyme into a thin inner layer or axial sheath, and a thicker outer layer. Polyps fully retractile within prominent calyces longitudinally and closely placed and at all sides of the branches. Calyces prominent, shelf-like or tubular, with prickly projecting spindles, longitudinally arranged, imbricate or not. Anthocodial sclerites mainly small spindles, in weakly differentiated transverse collaret and points below the tentacles, or just with some sclerites scattered along the neck zone of the polyp. Sclerites of the outer coenenchyme mostly long, unilateral spinous spindles, often massive, sculptured on inner surface by crowded complex tubercles and on outer surface by simple spines or prickles, and in some species with a few more or less prominent coarse, prickly projections. Axial sheath composed of capstans, spindles, or oval forms. Sclerite colours white, various hues of yellow, amber, orange, purple and red. Anthocodials with lower colour hues.

###### Distribution.

From Cape Hatteras, North Carolina to Brazil, including Bahamas, Greater and Lesser Antilles, and Caribbean islands (Bayer 1961); in the eastern Pacific from southern California to Peru. The genus occurs at depths down to 200 m, but normally found less than 100 m. *Muricea midas* Bayer, 1959 is the deepest record for the genus in the western Atlantic, 146 m (Bayer 1959); and *Muricea galapagensis* Deichmann, 1941 in the eastern Pacific, 91 m.

###### Remarks.

Colony shape and branching patterns are variable among *Muricea* species. The shape of calyces shelf-like or tubular, and related features as being imbricate or sparse show many intermediate forms. In the tubular-calyces species group the apical branches show a closer arrangement of calyces and smaller projecting angles in respect to the branch than at the lower branches. Therefore, the strongest character that separates *Muricea* from other genera is the type of sclerites.

##### Muricea acervata

Taxon classificationAnimaliaAlcyonaceaPlexauridae

Verrill, 1866

Figures 1 , 2

Muricea acervata Verrill, 1866: 327–328; Rossi 1955; Harden 1979: 142.
Muricea (Eumuricea) acervata Verrill, 1869a: 419–421.
Eumuricea acervata Kükenthal, 1924: 143.

###### Material.

Holotype: YPM 1791 (figured specimen), dry, Bay of Panamá, Panamá, F.H. Bradley, 1866, no more data. Schizotype: USNM 1130758 (donated by YPM).

###### Description.

**Figure 1.**
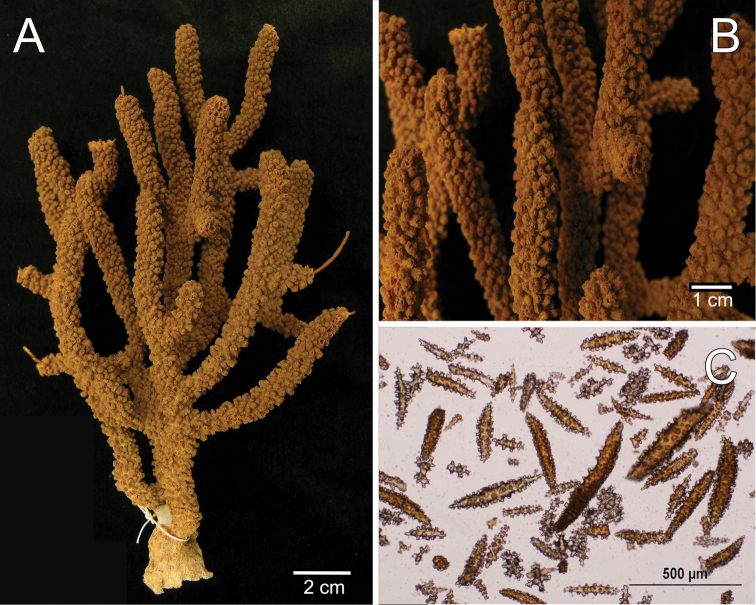
*Muricea acervata* Verrill, 1866 YPM 1791. **A** Colony **B** Detail of branches **C** Sclerites, light micrograph.

**Figure 2.**
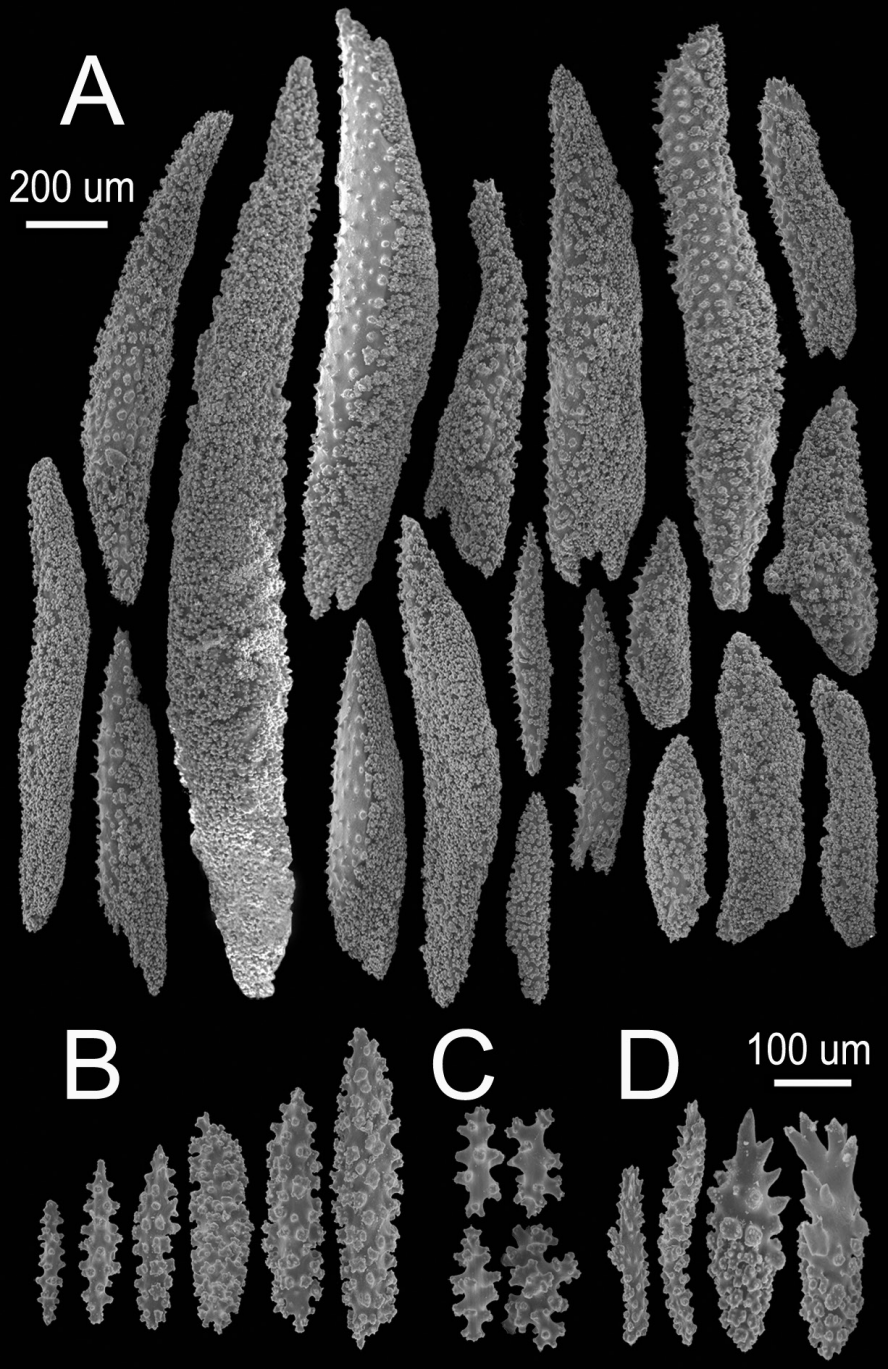
*Muricea acervata* Verrill, 1866 YPM 1791. **A** Calycular and coenenchymal spindles **B, C** Axial sheath spindles and radiates **D** Anthocodial sclerites.

The holotype is a 20 cm tall and 12 cm wide colony, the branching is lateral, almost in one plane (Fig. 1A) candelabrum-like. All branches are thick and rigid with almost the same diameter, 7–8 mm, from base to top. Two main branches, subdivide from a 2 cm long stem in secondary branches that remain unbranched up to the top of the colony, or subdivide up to 3 times producing branchlets of almost the same diameter. The branches are up to 20 mm apart, branch at angles of 45°–90°, and curve upwards, with blunt tips. Undivided terminal ends are up to 7 mm in diameter and 70 mm long (Fig. 1A–B). A vestige of the holdfast remains at the base of the stem. Axes are amber at the tips and darker at the base. Calyces are uniformly crowding the branches, close together, about 21 calyces/cm. They are up to 2.50 mm long and about the same in width, 1.8–2.0 mm. The rounded, small calyx apertures contain remains of anthocodial sclerites. The anthocodia are retracted and the eight projections of the calyces close over them. They are separated by slightly sunken grooves, which show an octoradiate star-like arrangement, that Verrill remarked as typical of this species (Verrill 1869a) (Fig. 1B). However, it is the normal condition of polyps in this genus, when retracted. The coenenchyme is thick compared with the other three species. The outer coenenchyme is composed basically by the same type of sclerites found in the calyx. They are spindles of several shapes, mostly unilateral spinous, curved, straight, with blunt or acute ends, or one acute end and the other bifurcate. They are 0.50–1.82 mm long and 0.15–0.28 mm wide (Fig. 2A), Verrill (1869a) reported spindles up to 2 mm long. They are of a light brownish to dark orange colour, some with the outer surface darker than the inner (Fig. 1C). The axial sheath is composed of pale yellow to colourless (Fig. 1C), warty elongated spindles 0.15–0.30 mm long and 0.060–0.085 mm wide (Fig. 2B), and irregular radiates, up to 0.24 mm long and 0.10 mm wide (Fig. 2C). Anthocodial sclerites are pale yellow, irregular warty rods with a spinulose end 0.25–0.30 mm long and 0.037–0.060 mm wide, and small torch-like clubs with a warty handle, measuring up to 0.28 mm long and 0.10 mm wide (Fig. 2D). The colour of the colony is brown.

###### Distribution.

Reported only from the type locality, Bay of Panamá. This species has not been found in our recent surveys along the Pacific coast of Panamá. No data available about the depth range.

###### Remarks.

This species was first mentioned by Verrill (1866) as *Muricea acervata* in 1869. It was transferred to the genus *Eumuricea* and properly described from just one specimen from Panamá that represents the holotype. The species is different from the others by the thicker coenenchyme, and especially the shorter calyces with a wider apical aperture that exposes the contracted polyps, which in the other species are hidden in the tubes. The dark orange colour of the calycular and coenenchymal sclerites is not present in the other species, which are of various hues of brown instead.

##### Muricea hispida

Taxon classificationAnimaliaAlcyonaceaPlexauridae

Verrill, 1866

Figures 3 , 4

Muricea hispida Verrill, 1866: 328; Harden 1979: 151–152.
Muricea (Eumuricea) hispida Verrill, 1869a: 422–423.
Eumuricea hispida Kükenthal, 1924: 151–152; Riess 1929: 398.

###### Material.

Lectotype (here designated): YPM 567, dry, Panamá, no depth given, F.H. Bradley, 1866. Paralectotype: YPM 1790, figured specimen in Verrill 1868, plate VII, fig 4, data as in the lectotype.

###### Other material.

USNM 49386 (erroneously identified as *Eumuricea hispida*), dry, Punta Arenas, Isla San Lucas, Golfo de Nicoya, Costa Rica, M. Valerio, 15 January 1930. USNM 34063 (erroneously identified as this species; it is a species of *Muricea*), dry, Panamá Bay, L.C. Cash, no more data. USNM 1016582, (erroneously identified as *Eumuricea hispida*), dry, Punta Paitilla, Panamá Bay, C.D. Ridder, 14 August 1976.

###### Description.

**Figure 3.**
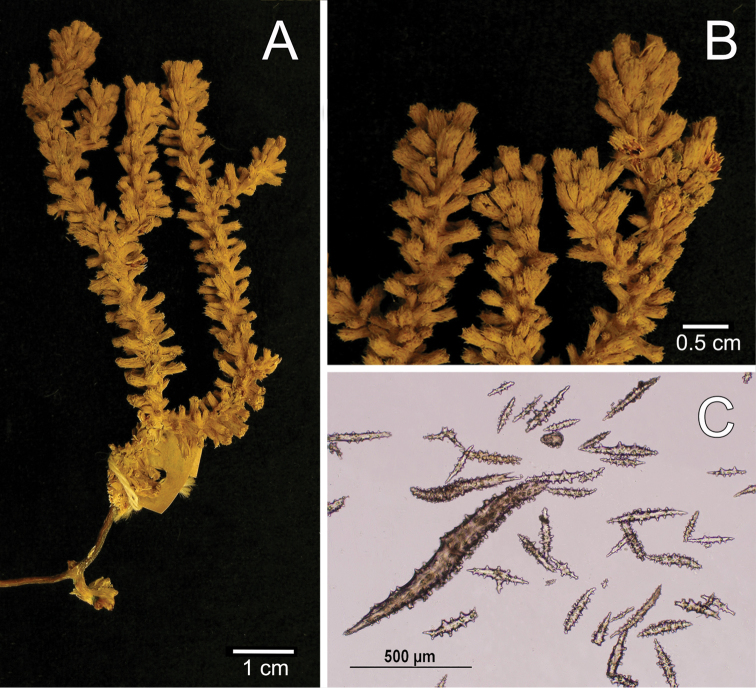
*Muricea hispida* Verrill, 1866 YPM 567 **A** Colony **B** Detail of branches **C** Sclerites, light micrograph.

**Figure 4.**
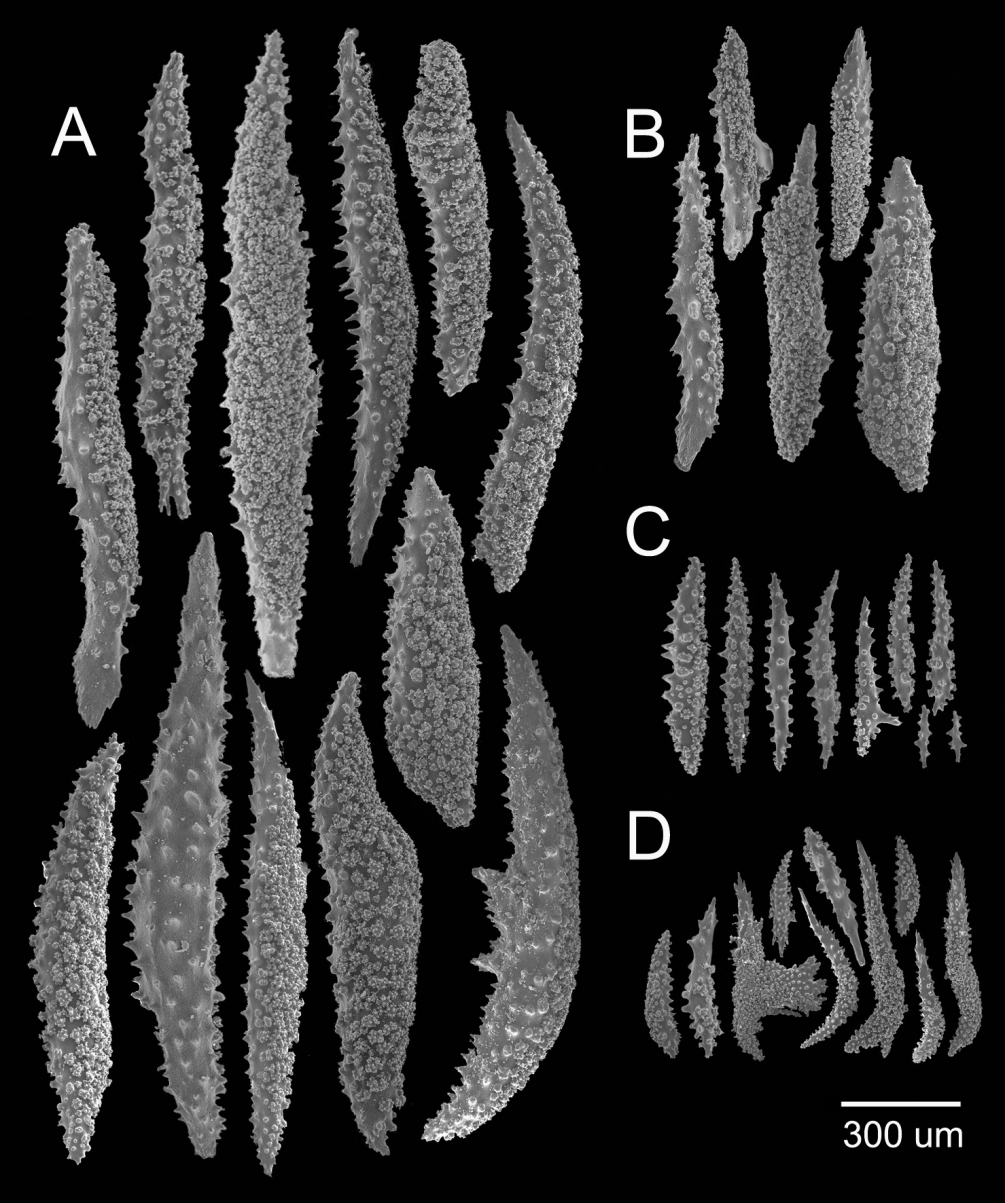
*Muricea hispida* Verrill, 1866 YPM 567. **A**, **B** Calycular and coenenchymal spindles **C** Axial sheath spindles **D** Anthocodial sclerites.

The lectotype is an 8.5 cm tall and 4 cm wide incomplete colony, branching is sparingly dichotomous (Fig. 3A). A short stem, 0.4 cm long, arises from a small remainder of the holdfast, and subdivides in two main branches deprived of coenenchyme, one of them is broken and the other subdivides in two secondary branches, 7–10 mm in diameter, that subdivide up to 4 times. All branches are almost the same diameter, with blunt, clavate tips. The branches are separated at distances of 0.6–5 cm and growing upwards at close angles of 30°–45°. Undivided terminal branches are up to 20 mm long, and 8 mm in diameter. The axes are dark brown at the base, and amber at the branchlets. The calyces are all around the branches, close together, about 14 calyces/cm. They are tubular and elongated reaching up to 3.5–4.0 mm long and up to 1.8–2.0 mm wide at the clavate tips; with projecting spines around the polyp apertures (Fig. 3B). The polyps are situated at the summit of the tubular calyces, the apertures are covered by anthocodial sclerites that represent what remained of the polyps. The coenenchyme is very thin, basically composed by the same type as the calyx sclerites. The outer coenenchyme and calycular spindles are unilateral spinous, spinulose on the outer surface and warty on the inner, 0.90–1.60 mm long and 0.14–0.20 mm wide, with acute ends, or one acute and the other blunt; others have stout, complex terminal spikes, 0.57–0.83 mm long and 0.10–0.14 mm wide (Fig. 4A, B). The axial sheath is composed of warty spindles with sparse warts and/or conical tubercles with acute tips; and irregular rods branched at one end, 0.13–0.56 mm long and 0.04–0.09 mm wide (Fig. 4C). The anthocodial sclerites are complex irregular branched forms, thorn scale-like with complex warts on the surface, sparse conical spines and/or with one spinulose end; irregular club-like spindles with warty handlers, straight or curved, and with spinulose, shaft-like heads. These sclerites are 0.26–0.70 mm long and 0.05–0.03 mm wide (Fig. 4D). All the sclerites are colourless (Fig. 3C). The colour of the colony is light brown.

###### Distribution.

Panamá, Bahía de Caraquéz, Ecuador (Riess 1929). No data available about the depth range.

###### Remarks.

This species was first mentioned by Verrill in1866, together with *Muricea acervata* with a minimal description. They both were properly described in 1869a. *Muricea hispida* was described from two specimen fragments from Panamá. *Muricea hispida* is similar to *Muricea squarrosa* and *Muricea tubigera*. These three species have long tubular calyces, similar colour and shape of the colonies. The main difference that separates them is the calyx length *Muricea tubigera* with the largest and *Muricea squarrosa* with the shortest (Table 1). The calyces in *Muricea hispida* are sharp and distally curved upwards with projecting spines beyond the calyx border as in *Muricea tubigera*, however, the latter has thinner, longer and more crowded calyces (Table 1). *Muricea tubigera* has the largest spindles, up to 2 mm long, in *Muricea hispida* up to 1.6 mm and in *Muricea squarrosa*, up to 1.3 mm (Table 1). *Muricea hispida* was misidentified in some collections, including the syntypes. For example, YPM 1636 listed as a syntype belongs to a different *Muricea* species, and other specimens, such as USNM 49386, 1016582 belong to *Muricea squarrosa*. We designate YPM 567as the lectotype of *Muricea hispida* to establish the identity of this species and avoid future misinterpretation.

**Table 1.** Comparative features of the eastern Pacific genus *Muricea* Lamouroux, 1821. Diameter of the branches is including calyces; size of the sclerites and other measurements are based on type material examined in this study. () Represents Verrill max size of sclerites. All measurements are given in mm.

Species	Colony colour	Colony shape	Length unbranched ends	Diameter of end branchlets	Coenenchyme	Calyx height	Calyx diameter	Calyx arrangement at branchlets	No. calyces/cm	Largest spindles	Axial sheath sclerites length range	Sclerite colours
*Muricea acervata*	b	cand	70	7	T	2.50	2	cl	21	1.8 (2)	0.15–0.30	lb, do, py, w
*Muricea hispida*	lb	bu	20	8	t	4	1.80	cl	14	1.6 (2.6)	0.13–0.56	w, c
*Muricea horrida	lb	bu	-	-	-	-	1.50	s	-	1.2	-	-
*Muricea squarrosa*	lb-b	bu	40	5	mt	2.6	1.75	s	14	1.3 (1.8)	0.14–0.30	py, lb, w, c
*Muricea tubigera*	lb	cand	70	8	mt	5	0.70	cl	26	2.0 (2.28)	0.12–0.46	w, c

* No type material available for this study, data given from Möbius 1861.

- No information available

calyx arrangement: cl, close; s, sparse

coenenchyme: t, thin, T, thick, mt, moderate thick

colour: b, brown, bi, bicoloured; c, colourless, transparent; do, dark orange; lb, light brown; py, pale yellow; w, whitish

colony shape: bu, bushy, ascending; cand, candelabrum, irregular dichotomous

##### Muricea horrida

Taxon classificationAnimaliaAlcyonaceaPlexauridae

Möbius, 1861 (sp. dubia)

Figure 5

Muricea horrida Möbius, 1861: 11–12; Kölliker 1865: 135; Harden 1979: 152.
Muricea (Eumuricea) horrida Verrill, 1869a: 423.
Eumuricea horrida Kükenthal, 1924: 151.

###### Material.

**Figure 5.**
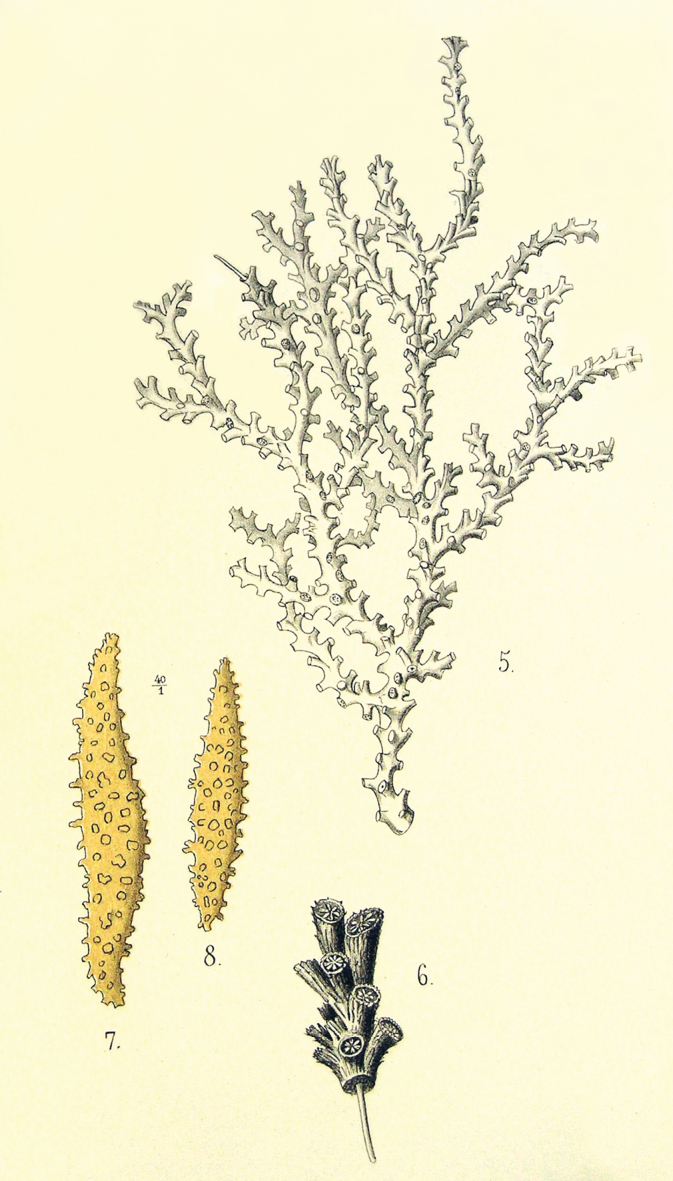
*Muricea horrida* Möbius, 1861. From plate 3, figs 5–8 (Möbius 1861).

**Figure 6.**
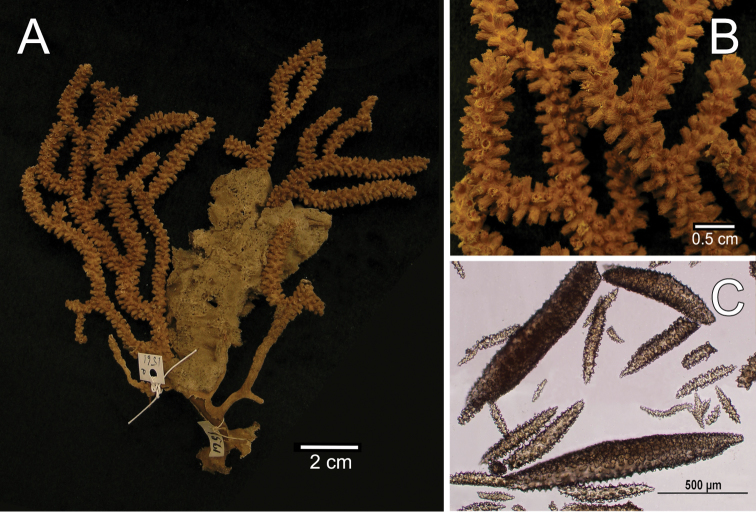
*Muricea squarrosa* Verrill, 1869a YPM 1561a. **A** Colony **B** Detail of branches **C** Sclerites, light micrograph.

**Figure 7.**
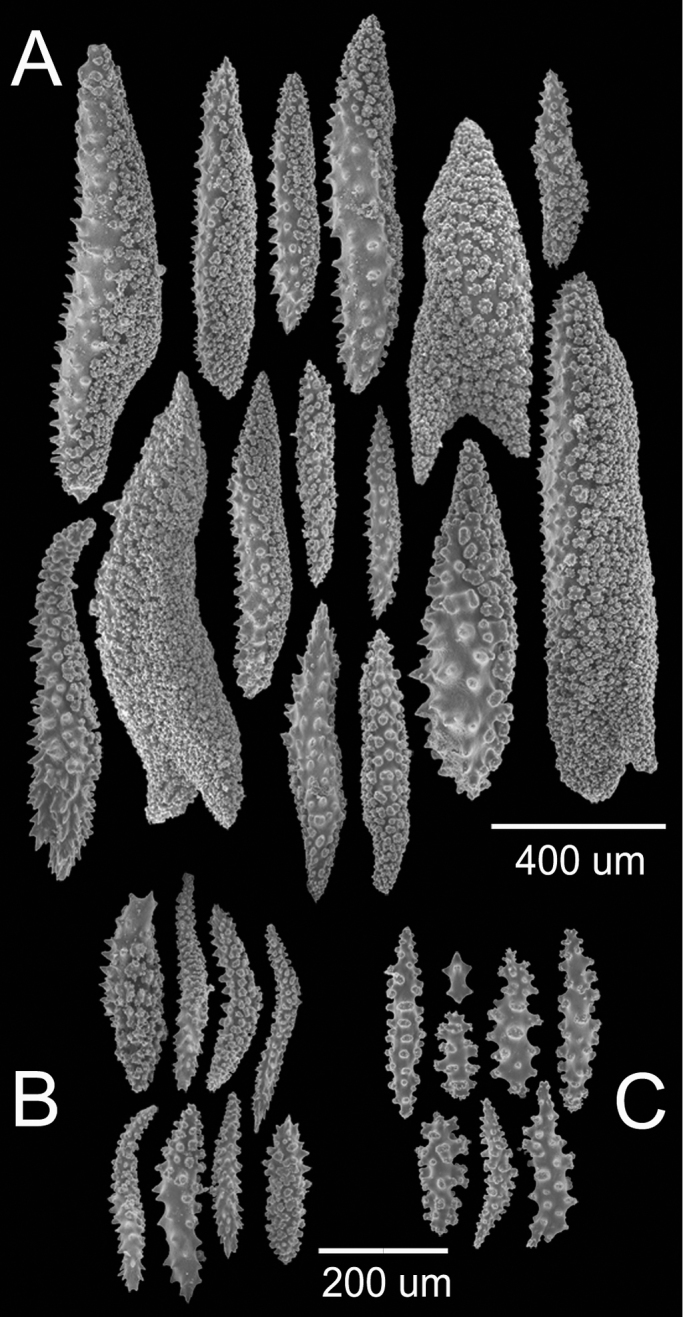
*Muricea squarrosa* Verrill, 1869a YPM 1561a. **A** Calycular and coenenchymal spindles **B** Anthocodial sclerites **C** Axial sheath spindles.

**Figure 8.**
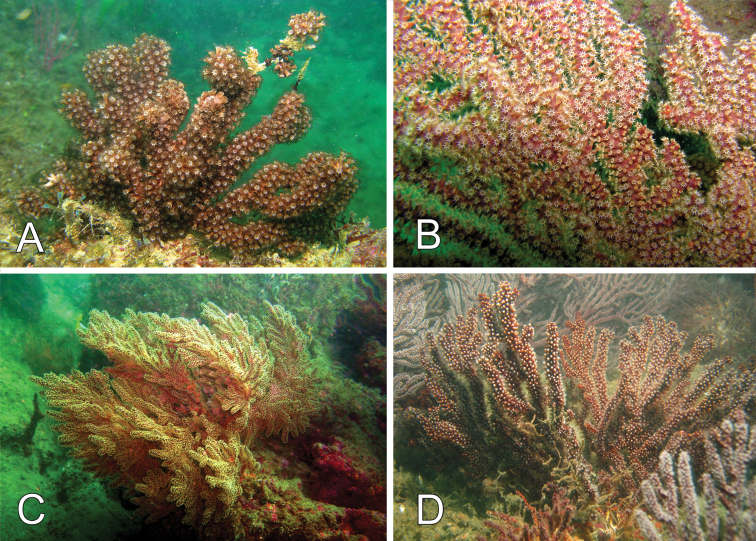
*Muricea squarrosa* Verrill, 1869a. *In situ* colonies, **A–C** Canoas de Punta Sal, Tumbes, Perú. Photograph by Yuri Hooker **D** Reserva de Producción Faunística Marino Costera Puntilla de Santa Elena, Ecuador. Photograph by Fernando Rivera.

Plate 3, figs 5–8 (Möbius 1861), no material available.

Holotype figured. According to Möbius (1861) the holotype was deposited in the Hamburg Museum (ZMH); however, the material was not housed there anymore (P. Stiewe and H. Roggenbuck, ZMH, pers. comm. 2011).

###### Description

**(after Möbius 1861 and Verrill 1869a).** The figured specimen is a fragment of a 20 cm tall and 22 cm wide colony with a thin, 6 cm diameter holdfast attached to a rock. The branching looks mostly dichotomous and starts close to the base (Fig. 5 [5]). The branches are closely placed and divergent, they subdivide at small angles and up to 6 times. All branches are about the same diameter with slightly tapered ends. Undivided terminal branches are short. The axes are brown at the base, and light yellow at the branchlets. The coenenchyme is granulose and brittle. The calyces are all around the branches, close together. They are mostly standing perpendicular to the branches, closer together and inclined upwards, at smaller angles, at the upper branchlets (Fig. 5[6]). They are tubular and elongated, up to 1.5 mm long with truncate tips. There is not enough information about the sclerites. They are straight or curved warty spindles reaching up to 1.2 mm long. They are yellow and seem asymmetric, perhaps unilateral spinose as for the genus, but from the drawings it is difficult to tell (Fig. 5[7, 8]). The colour of the colony is light brown.

###### Distribution.

Reported for Perú, the type locality.

###### Remarks.

According to Kükenthal (1924, in key) *Muricea horrida* differs from *Muricea squarrosa* in having shorter coenenchymal sclerites. Möbius (1861) description and illustration show a species that is similar to *Muricea squarrosa* from Perú. *Muricea squarrosa* is a common species in Perú. We did not find another similar species, a possible *Muricea horrida*, in the UPCH octocoral collection that is very comprehensive and well documented. It is indeed possible that *Muricea squarrosa* is a synonymous of *Muricea horrida*; however, without a specimen to analyse we prefer to keep the status of *Muricea horrida* as dubious.

##### Muricea squarrosa

Taxon classificationAnimaliaAlcyonaceaPlexauridae

Verrill, 1869

Figures 6 , 7 , 8

Muricea (Eumuricea) squarrosa Verrill, 1869a: 423–424.
Eumuricea squarrosa Kükenthal, 1924: 159.
Muricea squarrosa Harden, 1979: 159–160.

###### Material.

Lectotype (here designated): YPM 1561a, dry (with sponge), Pearl Islands, Panamá, F.H. Bradley, 1866, no further data; YPM 1563 [fragment of lectotype, possible figured specimen (Verrill 1869a)]. Paralectotypes: MCZ 4975; MCZ 7017; USNM 33592 (YPM 1561); YPM 1561b-d, YPM 566, data as for the lectotype. YPM 1636 (previously identified as *Eumuricea hispida*), ethanol preserved, Pearl Islands, F.H. Bradley, 1866, no further data.

###### Other material.

COSTA RICA: UCR 587, dry, Pitaya Beach, Guanacaste, Pacific coast, Costa Rica, 20–23 m, J. Cortés, 16 June, 1991; UCR 1742, ethanol preserved, Bajo Negro, Marino Ballena National Park, 25 m, O. Breedy,13 April 2008; UCR 2261, ethanol preserved, Isla Larga Oeste, Manuel Antonio National Park, 19 m, O. Breedy and H. Guzman, 6 February 2012; UCR 2262, ethanol preserved, Isla Larga, Manuel Antonio National Park, 25 m, O. Breedy and H. Guzman, 7 February 2012; UCR 2396, ethanol preserved, Marino Ballena National Park, 25 m, O. Breedy, 27 April 2002; UCR 2410; 2414, ethanol preserved, La Danta, Santa Elena Bay, 35 m, O. Breedy and Minor Lara, 10 August 2014; UCR 2418–2419, ethanol preserved, Bajo Mixta, Golfo Dulce, 21 m, O. Breedy and H. Guzman, 7 February 2009 ECUADOR: IIN 25, dry, Bajo Lunes, Reserva de Producción Faunística Marino Costera Puntilla de Santa Elena, 18 m, P. Martínez, F. Rivera, R. Nabot and O. Breedy, 21 July 2010; IIN 47, dry, Gigima, Reserva de Producción Faunística Marino Costera Puntilla de Santa Elena, 14 m, P. Martínez, F. Rivera, R. Nabot and O. Breedy, 22 July 2010. PANAMÁ: STRI 561, 563, 569–571, ethanol preserved, Islas Viudas, Chiriquí Gulf, Panamá, 20 m, H. Guzman, 18 April 2003; STRI 867–868, ethanol preserve, Achotines, Chiriquí Gulf, 10 m, H. Guzman, 5 May 2004; STRI 575A, ethanol preserved, Isla Saboga, 1–5 m, H. Guzman, 14 December 2001. PERÚ: UPCH-CZA 280, 284, 291, 296, 298, 302, 411, dry, Canoas de Punta Sal, Tumbes, 10–13 m, Y. Hooker, 2 July 2011; UPCH-CZA 410, Cabo Blanco, Piura, 10–13 m, Y. Hooker, 13 August 2012.

###### Description.

The lectotype is a 14 cm tall and 12 cm wide colony, flabellate, spreading in one plane. It has a sponge attached to the main branches (Fig. 6A). The branching is mostly dichotomous. A short stem, 0.4 cm long, 60 mm diameter, arises from an irregular holdfast, 23 mm in diameter, covered by a layer of coenenchyme, but deprived of calyces. The stem subdivides in two main branches that produce secondary branches subdividing up to 3 times. All branches are about the same in diameter, 40–80 mm (including calyces), with tapered ends. The branches are separated at distances of 0.5–6 cm and spread at small angles and bend upwards in a curve. The branchlets are situated almost perpendicular to the main branch. Undivided terminal branches are up to 40 mm long. The axes are brown at the base, and lighter at the branchlets. The calyces are all around the branches, close together, about 14 calyces/cm. They are mostly directed perpendicular to the branches, but also incline upwards at small angles (Fig. 6B). They are tubular and elongated, up to 2.6 mm long and up to 1.75 mm wide with clavate tips, between the larger calyces there are a number of smaller ones (Fig. 6B). The remains of the polyps are at the summit of the tubular calyces, the apertures are covered by anthocodial sclerites. The coenenchyme has a few layers of sclerites and is basically composed of the same types as the calyx spindles. They are straight or with a slight curvature. They are mostly acute, but can have one end blunt or lobed. They are unilateral spinous with the inner side with complex warts, crowded together so much that their processes anastomose, while on the outer side there are less and sparse spines. Some calycular spindles are club-like with warty elongated handles, straight or curved, and various types of head arrangement, from few conical spines to sharp and long spines crowding the upper part or along the outer side of the sclerite; they have stout terminal spikes (Fig. 7A). The spindles reach up to 1.3 mm long and 0.23 mm wide (Fig. 7A), Verrill (1869a) reported spindles up to 1.8 mm long. They are of a dull yellow to a light brownish colour. The axial sheath is composed of whitish and colourless, tuberculate spindles, 0.14–0.30 mm long and 0.03–0.075 mm wide (Fig. 7C) and irregular forms with- the same range of size, and immature forms 0.06–0.07 mm long and 0.015–0.02 mm wide. The anthocodial sclerites are of a pale yellow colour to colourless, mostly club-like as described for the calycular spindles, but shorter (Fig. 7B). The colour of the colony light brown.

###### Variability.

The other material examined is very consistent with the lectotype, variation is basically in the number of branches and size of the colonies. The largest colony measured was a specimen from Perú reaching 35 cm tall and 30 cm wide (Fig. 8C, *in situ*). The colony branching is abundant in some colonies (Fig. 8C, D). The colour of the colonies when alive is reddish brown (Fig. 8A–D). This colour fades in dry or ethanol preserved colonies. Fresh collected colonies turn the alcohol into a dark brownish colour. The polyps are pale yellow to whitish (Fig. 8A–D).

###### Distribution.

Panamá: Gulf of Chiriquí, Pearl Islands, 10–20 m. Costa Rica: Nicoya Gulf, Santa Elena Peninsula, Marino Ballena National Park, Golfo Dulce, from 25–40 m. Colombia: Málaga Bay (Prahl et al. 1986, specimens in CRBMco). Ecuador: Puntilla de Santa Elena, Salinas 18–20 m. Perú: Cabo Blanco, Canoas de Punta Sal, 10–13 m deep. Nicaragua: La Flor, Hueco de Diego, South Pacific, 2–5 m. The species has a wide bathymetric range from 2 m to 40 m, the deepest range being found in Costa Rica.

###### Remarks.

This species was described by Verrill (1869a) with specimens from Pearl Islands without a holotype designation and appropriate illustrations. We designate YPM 1561a as the lectotype of *Muricea squarrosa* to establish the identity of this species and avoid future misinterpretation.

The main difference to separate this species from *Muricea hispida* and *Muricea tubigera* is that the calyces are shorter and more distantly placed. Other differences were discussed above (under *Muricea hispida*).

##### Muricea tubigera

Taxon classificationAnimaliaAlcyonaceaPlexauridae

Verrill, 1869

Figures 9 , 10

Muricea (Eumuricea) tubigera Verrill, 1869a: 421–422.
Eumuricea tubigera Kükenthal, 1924: 150.

###### Material.

Holotype: YPM 807, dry, figured specimen, Pearl Islands, Panamá, low tide, F.H. Bradley, 1866.

###### Description.

**Figure 9.**
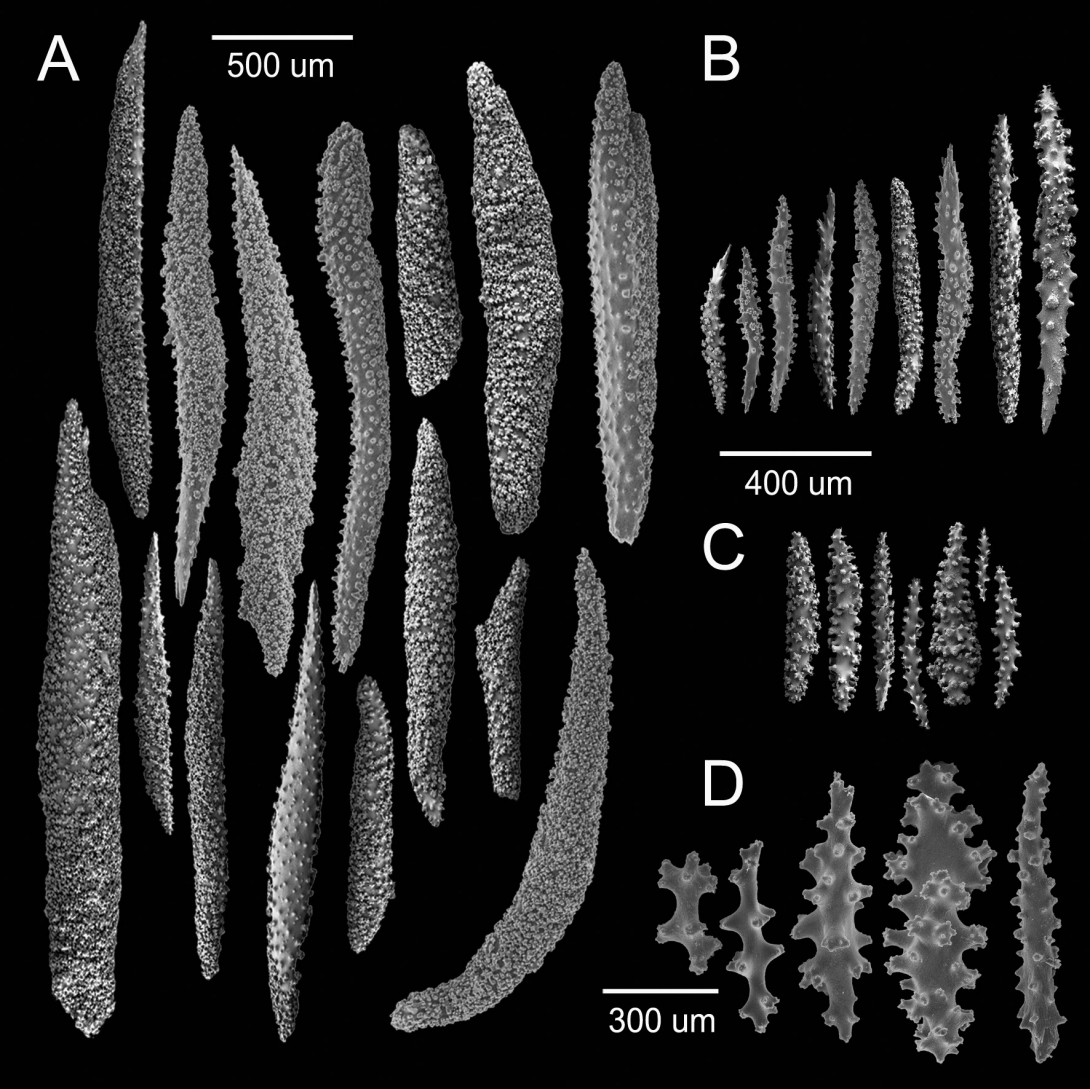
*Muricea tubigera* Verrill, 1869a YPM 807. **A** Colony **B** Detail of branches **C** Sclerites, light micrograph.

**Figure 10.**
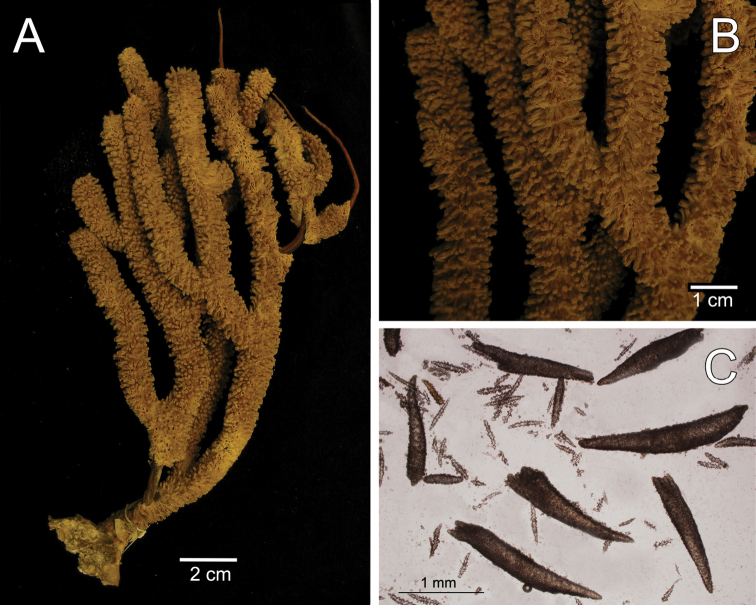
*Muricea tubigera* Verrill, 1869a YPM 807. **A, B** Calycular and coenenchymal spindles **C, D** Axial sheath spindles.

The holotype is a 17 cm tall and 10 cm wide stout and rigid colony, branching mostly dichotomous (Fig. 9A). A short stem, 1 cm in diameter, 1.5 cm long, arises from an oval 3 cm diameter holdfast, and subdivides in two main branches, 0.8–1.2 mm diameter, that fork producing secondary branches that subdivide up to 3 times. All branches are almost the same diameter with blunt, clavate tips. The branches are at distances of 2–7 cm apart and stick upwards at small angles of 30°–35°. Undivided terminal branches are up to 70 mm long, and 7–8 mm in diameter. The axes are dark brown. The calyces are uniformly crowding the branches, close together, about 26 calyces/cm. They are tubular, slender and elongated, up to 5 mm long and up to 0.75 mm wide, with clavate summits. The borders of the calyces are surrounded by long, slender and sharp spindles that project from the surface giving a prickly appearance to the branches (Fig. 9B). What remains of the polyps is at the summit of the elevated calyces, the apertures are covered by anthocodial sclerites and some calyx sclerites. The coenenchyme is of a few layers of sclerites, basically of the same types as the calyx spindles. They are mostly unilateral spinous spindles, large, slender, with sharp, blunt or bifurcated ends, some are spinulose on the outer surface and tuberculate on the inner, measuring 0.80–2.0 mm long and 0.07–0.30 mm wide (Fig. 10A). The calyx wall is mostly formed by warty, slender rods with one end acute and the other with long complex spines These sclerites are 0.435–0.76 mm long and 0.50–0.65 mm wide, they can have conic spines on the outer side of the sclerite and sparse warts on the inner side (Fig. 10 B). Verrill (1869a) reported a maximum size of 2.34 mm long. The axial sheath is composed of warty spindles (Fig. 10C) and tuberculate radiates, 0.12–0.46 mm long and 0.1–0.4 mm wide (Fig. 10D). All sclerites are whitish to colourless (Fig. 9C). The colour of the colony is light brown.

###### Distribution.

Reported only from the type locality, Pearl Islands, Panamá. This species has not been found in our recent surveys along the Pacific coast of Panamá.

###### Remarks.

Verrill (1869a) described this species with one specimen that constitutes the holotype. The very long and slender calyces of this species, the sharper spindles and the thickness of the branches separate this species from the others (Table 1).

##### Genus Swiftia

Taxon classificationAnimaliaAlcyonaceaPlexauridae

Duchassaing & Michelotti, 1864

Figure 12

Swiftia Synonymy in Breedy et al. (2015)

###### Diagnosis.

Colonies branching mostly in one plane, fan-like, dichotomous, pinnate-like, or unbranched. Branches mostly free or with some anastomosing. Polyp mounds conical, prominent, or slightly raised, scattered or crowded, usually biserial and with two opposed polyp mounds at the tip of the branches. Coenenchyme usually thin. Coenenchymal sclerites mainly capstans, radiates and spindles. Thin, sharp and elongated spindles concentrated in the polyp mounds. Anthocodiae with points arrangements of bar-like rods straight or curved, frequently long. Collaret absent or of a few bar-like rods. Axis horny and flexible. Colour of the colonies red, orange, pink, or white.

###### Type species.

*Gorgonia exserta* Ellis & Solander, 1786, by monotypy.

##### Swiftia pusilla

Taxon classificationAnimaliaAlcyonaceaPlexauridae

(Nutting, 1909) comb. n.

Figure 11

Eumuricea pusilla Nutting, 1909: 718–719; Kükenthal 1924: 152.

###### Material.

Holotype. USNM 25430, ethanol/dry preserved, Point Loma, San Diego, California, Albatross R/V, California Coast Expedition, 166–177 m, 15 May 1904.

###### Description

(after Nutting 1909: 718). The holotype was a small, roughly flabellate colony, 37 mm long, branching in an irregular manner. The main stem gives off four alternate branches at irregular intervals, the two longest being 13 mm apart. The calyces are low rounded domes, about 1 mm long and 2 mm wide, separated about 2.5 mm from summit to summit. The polyps are completely retracted. “The calycular walls are covered with very hispid spicules (sclerites), which have their edges somewhat overlapping and are, in general, disposed transversely rather than otherwise”. Nutting reports the presence of a collaret and tentacles armed with sharp spindle-shaped sclerites longitudinally arranged, but *in chevron* at the base of the tentacles. Other type of sclerites are asymmetrical spindles with irregular sharp edges and processes, various types of clubs, scales, stars and double stars. The colour of the colony is whitish to gray.

###### Distribution.

Reported for the type locality Point Loma, California.

###### Remarks.

**Figure 11.**
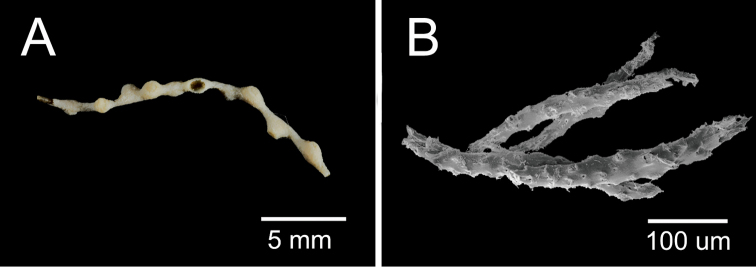
*Swiftia pusilla* (Nutting, 1909). **A** Fragment of the holotype **B** SEM sclerites. Photographs by S. Cairns (USNM).

What remain from the holotype are small pieces of branches: two fragments, 16 mm and 12 mm long, the former with 9 polyps, the latter with 5 (pers. comm. S. Cairns) (Fig. 11A). Nutting’s illustrations (1909, PL. LXXXVIII) show some fragments of a thin colony. The sclerites are almost disintegrated, SEMs obtained by S. Cairns (USNM) show spindles as the prevailing type of sclerites (Fig. 11B). It is not possible to confirm the other types of sclerites described by Nutting (1909) and his description is fairly general. However, the characteristics that we could analyse of the species fit with the genus *Swiftia*. For this reason, we herein propose the genus *Swiftia* as a more accurate alternative for the species (Table 2).

**Table 2.** Proposed genera for species misplaced in the genus *Eumuricea* Verrill, 1869.

Species	Original author	Author *	Actual status	Proposed status	Distribution	Depth (m)
*Eumuricea atlantica*	Riess, 1929	Bayer 1961	*Muricea* Lamouroux		Tortugas, Kingston, Jamaica, Caribbean sea	18
*Eumuricea pusilla*	Nutting, 1909	herein	*Eumuricea*	*Swiftia* Duchassaing & Michelotti, 1864	Point Loma, California, Pacific Ocean	176
*Eumuricea ramosa*	Thomson & Simpson, 1909	herein	*Eumuricea*	*Astrogorgia* Verrill, 1868	Andaman sea, Indian Ocean	83–494
*Eumuricea rigida*	Thomson, 1927	Ofwegen, L.P. van 2014	*Thesea*		Along Monaco, Western Atlantic	1732
*Eumuricea rugosa*	Thomson, 1927	Grasshoff, 1992	*Leptogorgia ruberrima*		Iles Cap Vert, Western Atlantic	91
*Eumuricea splendens*	Thomson & Simpson, 1909	herein	*Eumuricea*	*Astrogorgia* Verrill, 1868	Marble Rock, Mergui Archipelago, Andaman sea, Indian Ocean	not given

* Author who transferred the original species to another genera or our new proposed genera for the species.

##### Genus Astrogorgia

Taxon classificationAnimaliaAlcyonaceaPlexauridae

Verrill, 1868

Astrogorgia Verrill, 1868b: 414; Verrill 1870: 77–78; Bayer 1981: 931 (in key); Grasshoff 1999: 38; 2000: 67; Fabricius and Alderslade 2001: 210–213; Hermanlimianto and Ofwegen 2006: 103.
Muricella Kükenthal, 1924: 169.
Acanthomuricea Fabricius & Alderslade, 2001: 212.

###### Type species.

*Astrogorgia sinensis* Verrill, 1868b by monotypy.

###### Diagnosis

[based on Grasshoff (2000), Fabricius and Alderslade (2001), Hermanlimianto and Ofwegen (2006)]. Colonies growing in one plane as open fans, with irregular lateral branching, never net-like. Polyps retractile into raised calyces, arranged in rows or all around the branches. Coenenchymal sclerites mostly spindles, straight, curved, branched, heavily ornamented with complex tubercles, and prickles; and smaller spindles and some capstans in the inner-coenenchyme. Anthocodiae with numerous flattened sclerites around the tentacle bases and up the tentacles in numerous oblique rows. Collaret does not occur. Colour of the colonies, various hues of red, orange, yellow, whitish or yellowish brown.

##### Astrogorgia splendens

Taxon classificationAnimaliaAlcyonaceaPlexauridae

(Thomson & Simpson, 1909) comb. n.

Figures 12 , 13

Eumuricea splendens Thomson & Simpson, 1909: 258–259.

###### Material.

Holotype: BM 1933.05.03.094, ethanol preserved, Marble Rock, Mergui Archipelago, Myanmar, Andaman Sea. No more data available.

###### Description

**Figure 12.**
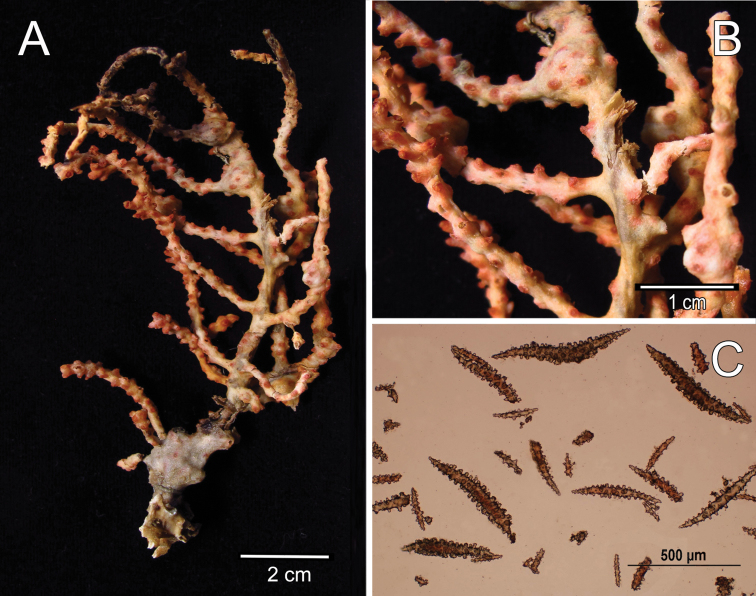
*Astrogorgia splendens* (Thomson & Simpson, 1909), BM 1933.05.03.094. **A** Colony **B** Detail of branches **C** Sclerites, light micrograph.

**Figure 13.**
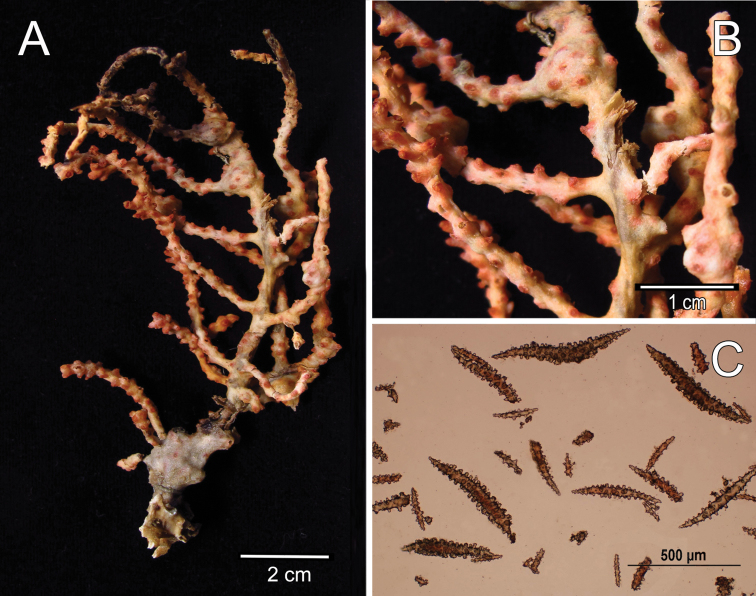
*Astrogorgia splendens* (Thomson & Simpson, 1909), BM 1933.05.03.094. SEM sclerites.

[see also Thomson and Simpson (1909)]. The holotype is a 9.5 cm tall and 6 cm wide colony. Several stems arise from a spreading holdfast but only one branch ramifies in two secondary branches, the others are broken close to the base (Fig. 13A, B) that is partially covered by a sponge. The branching is lateral and irregular, predominantly in one plane. Secondary branches subdivide up to 7 times upwards at small angles. Free end branches reach up to 3.5 cm long. The axis is horny and of a light brown colour. The polyps are prominent and distributed longitudinally in two rows at the base of the main branches, but more irregularly and crowded at the upper parts. The calyces are prominent up to 2 mm in diameter and up to 1.5 mm high (Fig. 12B). The anthocodial sclerites are arranged in collaret and points, “en chevron” at the base of the tentacles. The anthocodiae are completely retractile and show an octoradiate star-like arrangement. The coenenchyme and calyces are composed of whitish and reddish sclerites (Fig. 12C). They are mostly warty spindles, straight, curved, and branched, mostly with acute ends, and ornamented with complex tubercles and prickles. These spindles measure 0.21–1.0 mm long and 0.046–0.16 wide (Fig. 13). The anthocodials are warty rods, 0.15–0.20 mm long and 0.03–0.06 mm wide (Fig. 12C). The colour of the colony is pale pink with reddish calyces.

###### Distribution.

From the type locality, Marbel Rock, Mergui Archipelago, Andaman Sea, Indian Ocean. No data available about the depth range.

###### Remarks.

The two species described in *Eumuricea* by Thomson and Simpson (1909) appear in the BM catalogue as species of the genus *Muricella*, *ramosa* and *splendens*. However, Fabricius and Alderslade (2001), and Grasshoff (1999) refer to the genus *Muricella* as being planar large fans, often net-like, and large, with thick coenenchyme. *Eumuricea splendens* sensu Thomson & Simpson (1909) is a small specimen 9.5 cm in height, and *Eumuricea ramosa* is supposedly a large specimen, both with thin coenenchyme, and without net-like colonies. The description and sizes of the sclerites given by the above authors for the genus *Muricella* do not fit these two species. Furthermore, Thomson and Simpson’s (1909) holotype of *Eumuricea splendens* does not agree with the characteristics of *Eumuricea*. Although Thomson and Simpson (1909), acknowledge some resemblance with *Eumuricea acervata*, the holotype does not have tubular calyces and does not show the characteristic unilateral spinous spindles of *Eumuricea*. The dominant types of sclerites are acute warty spindles and variations. Therefore, we propose to transfer this species to the genus *Astrogorgia*.

##### Astrogorgia ramosa

Taxon classificationAnimaliaAlcyonaceaPlexauridae

(Thomson & Simpson, 1909) comb. n.

Eumuricea ramosa Thomson & Simpson, 1909: 260–261.

###### Material.

None available.

###### Description

[based on Thomson and Simpson (1909)]. Thomson and Simpson (1909) described a colony 23 cm tall and 30 cm wide. The branching is irregular, predominantly in one plane. The main stem is sinuous, about 8 mm in diameter arising from a conical holdfast. The branches are tapered at the ends, and the twigs are of almost the same thickness throughout, some are clavate. The axis is horny, composed of thin sheets of gorgonian. The coenenchyme is moderately thin. It is composed of colourless sclerites irregularly arranged at the lower part of the branches and more longitudinally placed at the twigs. The polyps are distributed all around the branches closer at the upper branches and more separated at the lower parts. The anthocodiae are completely retractile into slightly elevated cones, 1 mm in height and 1 mm in diameter at the base. The anthocodial sclerites are arranged in eight distinct groups “en chevron” at the base of the tentacles with projecting teeth around the oral aperture. The coenenchymal sclerites are spindles, straight, curved or S-shaped, with acute or blunt ends, with the surface covered by warts, they measure 0.4–1.5 mm long and 0.075–0.17 wide. The anthocodiae are club-shaped, with warty heads and smooth handles, 0.3–0.6 mm long and 0.05–0.1 mm wide. The colour of the colony is a greyish white.

###### Distribution.

From the type locality, Andaman sea, Indian Ocean, 83–494 m in depth.

###### Remarks.

We only have a few drawings of sclerites of this species from Thomson and Simpson (1909: Plate VIII. Fig. 15). The type material was not available for analysis, however, the sclerite drawings, the depth range and the geographic distribution of this species is not consistent with the genus *Eumuricea*. Considering that the Thomson and Simpson’s (1909) description and illustrations of this species and *Eumuricea splendens* closely agree; we also propose, with some caution, to transfer *Eumuricea ramosa* to the genus *Astrogorgia* (Table 2).

#### Family Gorgoniidae Lamouroux, 1812

##### Leptogorgia ruberrima

Taxon classificationAnimaliaAlcyonaceaGorgoniidae

(W. Koch, 1886)

Figure 14

Gorgonia ruberrima W. Koch, 1886: 14–18.
Eumuricea rugosa Thomson 1927: 48.
Leptogorgia monodi Stiasny 1937: 309.
Leptogorgia ruberrima Stiasny, 1940: 361; Grasshoff 1988: 111; 1992: 72 (synonymy according to Grasshoff 1992).

###### Material.

Holotype: BM 1933.03.13.024, fragment, ethanol preserved, Campagne 1901, Stn. 1203: 15°54’ N, 22°54'45"E, Iles du Cap Vert, 91 m, 18 August 1901.

###### Description

**Figure 14.**
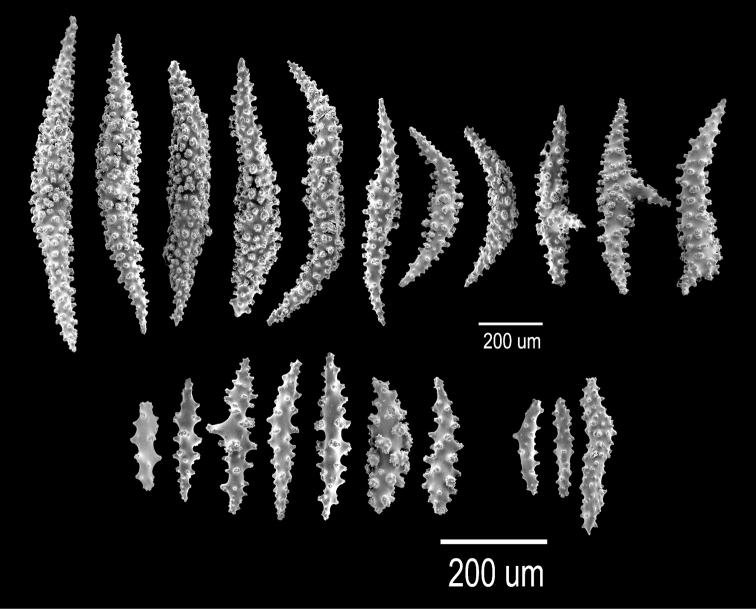
*Leptogorgia ruberrima* (W. Koch, 1886), BM 1933-03-13-024. **A** Fragment of the holotype **B** Sclerites, light micrograph **C** SEM sclerites.

**(see also Thomson 1927).** The holotype is a bright red fragment, 3 cm long and 3 cm wide (Fig. 14A). Thompson (1927) described a 15.5 cm tall colony. The branches are 2 mm in diameter. The axis is amber. The calyces are cones projecting up to 0.75 mm high and about 1 mm in diameter. They are placed all around the branches about 1 mm apart. The coenenchymal sclerites are red and basically warty spindles with acute ends, straight or curved, 0.2 mm–0.32 mm long and 0.065 mm–0.087 mm wide, and radiates 0.10–0.20 mm long and 0.04 mm–0.045 mm wide (Fig. 14B–C). Anthocodial sclerites are flat orange rods in an irregular point and collaret formation. They are 0.050 mm–0.15 mm long, with lobed or smooth borders (Fig. 14B). The sizes of sclerites given by Thomson (1927) are smaller than the ones we analysed in the holotype fragment. The colour of the colony is bright red.

###### Distribution.

Reported from the scientific campaigns of Prince Albert 1st de Monaco in 1901, Station 1203, along Iles du Cap Vert, 15°54’ N, 22°54'45"E, Western Atlantic.

###### Remarks.

Thomson (1927) described two species of *Eumuricea*, *Eumuricea rigida* and *Eumuricea rugosa*but neither of these fit in the genus *Eumuricea*. The former was transferred to *Thesea* (Table 2) by Ofwegen (2014), and the latter does not show the characteristic spheroid plate-like sclerites of *Thesea* in the outer coenenchyme. The coenenchyme of *Eumuricea rugosa* is composed of acute, elongated spindles instead. We confirm the finding of Grasshoff (1992) that this is nothing other than *Leptogorgia ruberrina* (W. Koch, 1886).

## Conclusions

Firstly, we conclude that Verrill’s genus *Eumuricea* was misinterpreted by former authors who erroneously assigned this genus to a diverse group of species. Secondly, that *Eumuricea* corresponds to a group of *Muricea* with tubular calyces as Verrill proposed, but because all other characteristics are found in *Muricea*, we do not consider the shape of the calyx as a sole character to separate this genus, since there are at least three more types of calyx structure within *Muricea*. And thirdly, the *Muricea* group with tubular calyces comprises four valid species *Muricea acervata*, *Muricea hispida*, *Muricea squarrosa* and *Muricea tubigera*, and a dubious one, *Muricea horrid*, reported for the eastern Pacific. It is intriguing that *Muricea squarrosa* is the only species in the genus that recently has been collected at various localities along the eastern Pacific. Although extensive surveys have been conducted along the Pacific coast of Panama and Peru (main type localities), the other species have not been found. Presently, we do not have data to support the idea of local extinction of species, but it is very likely that changing oceanographic conditions could have affected octocoral diversity. Perhaps more survey effort along the eastern Pacific would give further information about this genus. Presently, we recommend taking into account the status of the species of *Eumuricea* for biodiversity records and assessments.

## Supplementary Material

XML Treatment for Muricea

XML Treatment for Muricea acervata

XML Treatment for Muricea hispida

XML Treatment for Muricea horrida

XML Treatment for Muricea squarrosa

XML Treatment for Muricea tubigera

XML Treatment for Swiftia

XML Treatment for Swiftia pusilla

XML Treatment for Astrogorgia

XML Treatment for Astrogorgia splendens

XML Treatment for Astrogorgia ramosa

XML Treatment for Leptogorgia ruberrima
