# Corrigenda: Breedy O, Guzman HM (2015) A revision of the genus *Muricea* Lamouroux, 1821 (Anthozoa, Octocorallia) in the eastern Pacific. Part I: *Eumuricea* Verrill, 1869 revisited. ZooKeys 537: 1–32. doi: 10.3897/zookeys.537.6025

**DOI:** 10.3897/zookeys.553.7471

**Published:** 2016-01-14

**Authors:** Odalisca Breedy, Hector M. Guzman

**Affiliations:** 1 Centro de Investigación en Estructuras Microscópicas, Centro de Investigación en Ciencias del Mar y Limnología, Universidad de Costa Rica. P.O. Box 11501-2060, Universidad de Costa Rica, San José, Costa Rica; 2 Smithsonian Tropical Research Institute, P.O. Box 0843-03092, Panama, Republic of Panama

Several errors came to our attention after our manuscript was published, which we address here. First, Figure 9 corresponds to the colour plate under Figure 10; Figure 10 corresponds to the SEM plate under Figure 9. Second, Figure 13 corresponds to the SEM plate under Figure 14. Third, Figure 14 is a new plate that was missing. Finally “Figure 12” that is written under **Genus *Swiftia* Duchassaing & Michelotti, 1864**, in page 21 is a typo.

The correct, whole Figures and captions are reproduced here below.

**Figure 9.**
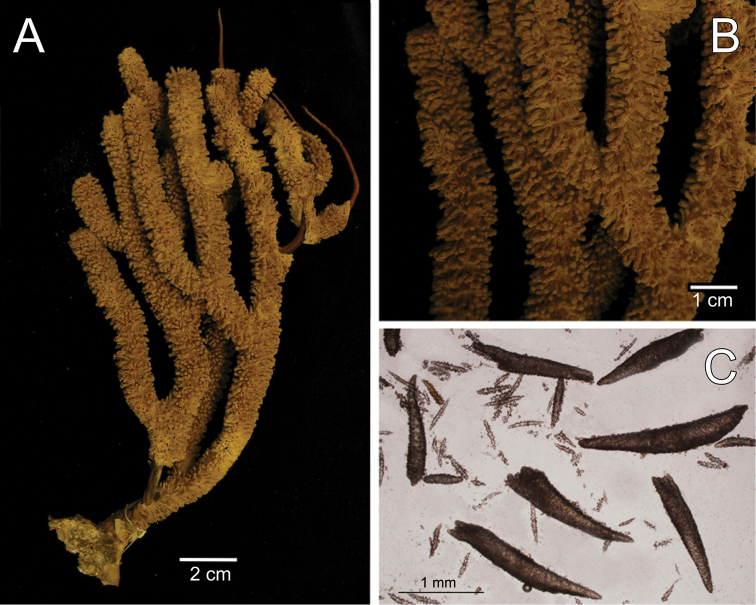
*
Muricea tubigera* Verrill, 1869a YPM 807. **A** Colony **B** Detail of branches **C** Sclerites, light micrograph.

**Figure 10.**
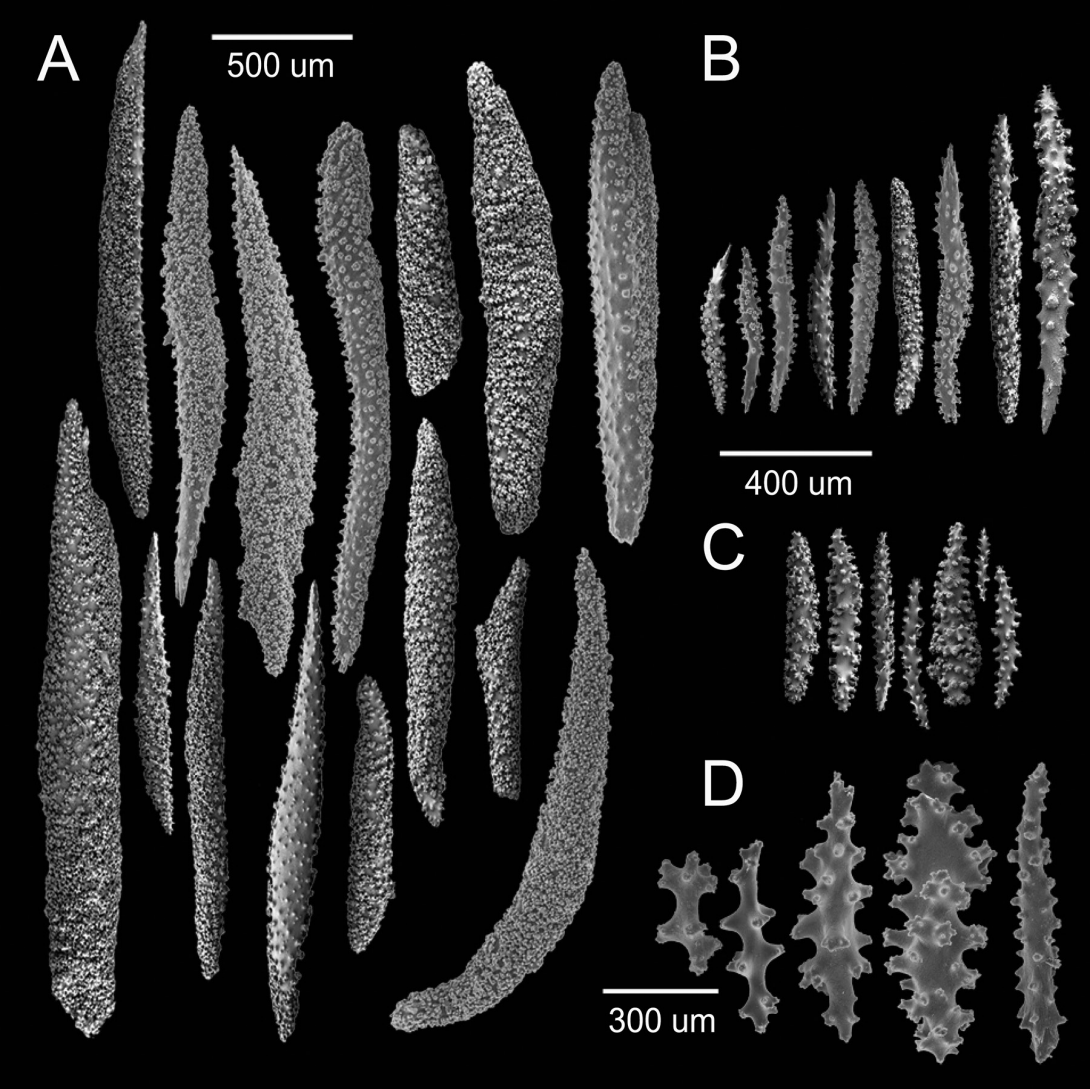
*
Muricea tubigera* Verrill, 1869a YPM 807. **A, B** Calycular and coenenchymal spindles **C, D** Axial sheath spindles.

**Figure 13.**
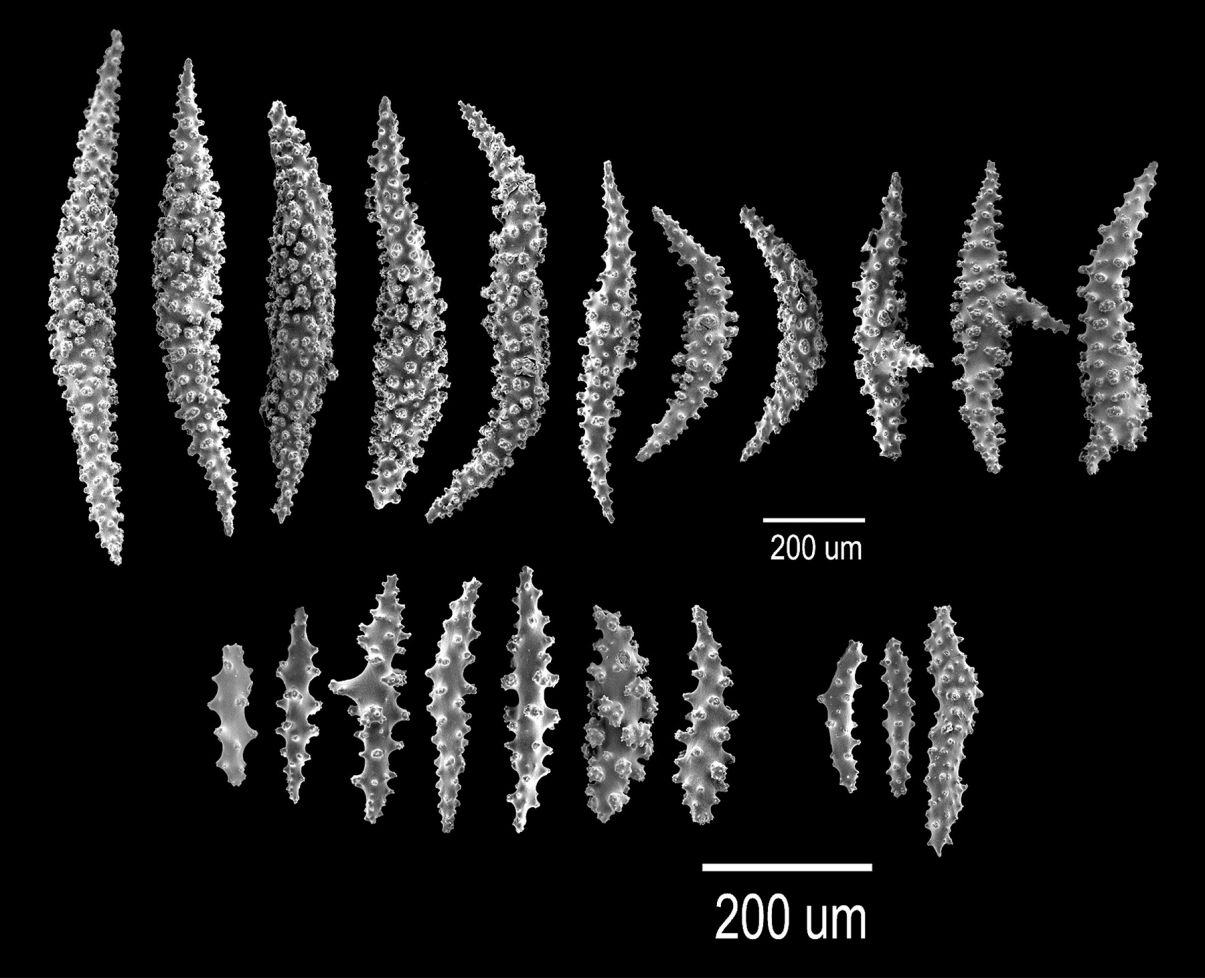
*
Astrogorgia splendens* (Thomson & Simpson, 1909), BM 1933.05.03.094. SEM sclerites.

**Figure 14.**
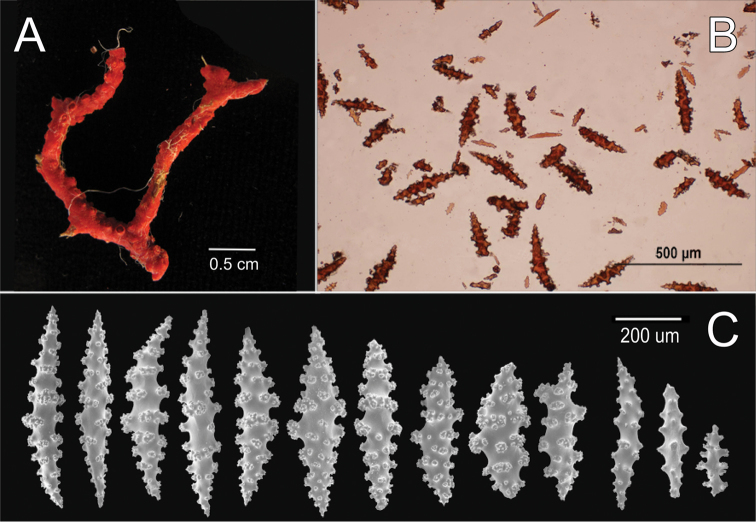
*
Leptogorgia ruberrima* (W. Koch, 1886), BM 1933-03-13-024. **A** Fragment of the holotype **B** Sclerites, light micrograph **C** SEM sclerites.

